# Addressing sexual health in oncology: perspectives and challenges for better care at a national level

**DOI:** 10.3332/ecancer.2024.1765

**Published:** 2024-09-13

**Authors:** Natalia Camejo, Camila Montenegro, Dahiana Amarillo, Cecilia Castillo, Gabriel Krygier

**Affiliations:** Clinical Oncology Service, Hospital de Clínicas Dr. Manuel Quintela, Montevideo 11600, Uruguay; a https://orcid.org/0000-0002-8684-0291; b https://orcid.org/0009-0004-6888-9544; c https://orcid.org/0000-0002-8615-8639; d https://orcid.org/0000-0002-0417-0512; e https://orcid.org/0000-0002-0518-1854

**Keywords:** sexual health, cancer survivors, communication barriers, knowledge, attitudes and health practice

## Abstract

**Introduction:**

The emotional impacts of oncological treatments can negatively affect sexual health and intimate relationships. Advances in cancer management have extended patient survival, underscoring the importance of addressing sexual health post-diagnosis.

**Objectives:**

To explore physicians’ practices regarding the approach to sexual health during oncological consultations; identifying barriers to addressing sexuality and assessing the need for sexual health training.

**Methods:**

An observational, cross-sectional study that assessed the management of sexual health by physicians involved in oncological treatment, using an anonymous questionnaire distributed via SurveyMonkey.

**Results:**

Of 133 physicians surveyed, 31.6% never or rarely address sexual health. Only 10.5% feel frequently prepared on this topic, while 24.8% almost never have the appropriate tools to address it. 97.7% of oncologists and 92.9% of otolaryngologists (ENTs) recognize the need for sexual health training. Sexual health was more frequently discussed among patients diagnosed with prostate, cervical and breast cancer, and less so among those with ENT, bladder and colorectal tumours. The approach was more frequent among patients treated with curative intent (77.4%) than those with palliative intent (5%). The main barriers identified were lack of training (46%), lack of time (39.8%) and patient discomfort (34.6%).

**Conclusion:**

The majority of professionals dealing with oncological patients do not address their sexual health, with the lack of training, lack of time and patient discomfort being the main barriers identified. However, 92% indicate a need for sexual health training, which could contribute to early intervention, strategy establishment and timely referral to specialists in the field.

## Introduction

The prolongation of survival of patients diagnosed with cancer due to new therapies highlights the need for a comprehensive and multidisciplinary approach, including sexual health. Sexual health, essential for the integral wellbeing of the human being, transcends the mere absence of disease or sexual dysfunction. Defined by the World Health Organisation, it encapsulates the state of physical, mental and social well-being in relation to sexuality. This conception encompasses a holistic and positive understanding, emphasizing not only the importance of pleasurable and safe sexual experiences, free from coercion, discrimination and violence, but also the respect, protection and full exercise of the sexual rights of each individual [1].

Sexual health is not limited only to sexual function, such as frequency, orgasmic capacity and desire, but also includes affectivity, the need for intimacy and the role of the partner. These aspects are fundamental to the emotional well-being and quality of life of oncology patients. Affectivity and intimacy allow for a deep emotional connection, while partner support and understanding are essential for coping with the challenges related to cancer and its treatments. It is crucial that healthcare professionals address these elements to provide comprehensive care that is sensitive to the needs of their patients [1–4].

From an inclusive and affirmative approach, the conceptualisation of sexual health transcends its multifaceted nature, encompassing dimensions that are essential for a comprehensive understanding of this construct:

1. Holistic wellness: Sexual health transcends the mere absence of disease or dysfunction, promoting a state of physical, emotional and social well-being closely linked to sexuality. This holistic approach highlights the importance of a full and satisfying sexual experience as a fundamental component of overall health.
2. Respect and safety: This aspect emphasizes the creation of safe and respectful environments, free of discrimination and violence, as fundamental to the healthy development of individual sexuality. Personal safety and mutual respect are imperative for healthy and non-coerced sexual expression.
3. Human rights: Sexual health is situated within the framework of sexual rights, which are considered extensions of basic human rights. This approach emphasizes the need to guarantee and protect these rights in order to facilitate the full realisation of sexual health for all individuals.
4. Universality across the life cycle: Recognising the importance of sexual health across all stages of life, this principle holds that sexual needs and rights are relevant and crucial from youth to old age, reflecting a life-cycle perspective.
5. Sexual diversity: This approach highlights the need to recognize, accept and value diversity in sexualities and forms of sexual expression, promoting a culture of inclusion and respect for all variants of sexual experience.
6. Influence of gender: Gender constructs-including norms, roles, expectations and associated power dynamics-play a critical role in sexual health. A thorough analysis of these factors is essential for effective sexual health promotion and protection.
7. Sociocultural context: The interpretation and approach to sexual health must be contextualised within the specific social, economic and political frameworks in which individuals live. This principle recognizes how intersectionality and multiple factors shape and affect people’s sexual experience [1].

In this context, sexual health is crucial to quality of life and overall health, including in oncology. Cancer treatments, while vital for cancer management, can have significant side effects on sexual health. These can manifest as alterations in sexual desire, difficulties with arousal and orgasm and an overall decrease in sexual satisfaction. Such changes may begin at diagnosis and persist, or even intensify, during treatment and beyond, negatively impacting the patient’s perception of well-being and quality of life. Consideration of these effects from the beginning of oncologic management, and throughout the treatment process, is critical to ensure comprehensive care that considers sexual well-being as a key component of overall health [2, 3].

In addition, not only treatments can have an influence, but also psychological and emotional disorders that arise after a cancer diagnosis, such as the appearance of fear, anxiety and anguish, which can affect sexual health and intimacy [4–6]. 

At least 40% of cancer patients experience alterations at the sexual level as a consequence of chemotherapy, radiotherapy, hormone therapy and surgery; which produce alterations at the physical level, such as alopecia, weight change and surgical scars, which lead to alterations in body image and have repercussions at the emotional level, making it difficult for the patient to enjoy sexual activity and feel sexually attractive [3, 4]. Among women who have survived cancer, frequent alterations in their sexual health have been identified, such as reduced sexual desire, vaginal dryness, pain during intercourse, obstacles to orgasm and premature menopause. These problems may be the consequence of hormonal treatments or surgical interventions in the pelvic area. Likewise, changes in physical appearance, such as those resulting from a mastectomy for breast cancer treatment, hair loss or weight changes, have a significant impact on the perception of sexual health [2–4]. Among men, especially those undergoing androgen suppression therapies, they report with higher incidence problems such as decreased sexual desire, erectile difficulties and urinary incontinence [7]. In addition, chemotherapy, especially those combinations involving multiple drugs, has been found to be linked to an increased prevalence of sexual problems among testicular cancer survivors [8].

Other aspects that can have an emotional influence, such as the patient’s or partner’s fear of generating pain or death during intimacy and the lack of privacy in patients who are hospitalised, negatively affect sexual functioning [9, 10]. Because of this, in oncology patients, sexual health is usually resignified, leaving the physical part in the background, prioritising intimacy, thus reinforcing the emotional connection with the partner [11].

There are multiple barriers to communication between health professionals and patients about sexual health that make it difficult to address sexual health in clinical practice, probably influenced by lack of knowledge, training and time, cultural and psychological factors of both patients and health care providers [12, 13].

This study seeks to investigate the approach to sexual health in oncology patients by the healthcare team at the national level, which will make it possible to implement actions to improve the quality of care.

### Objective

To explore the practices of medical professionals (gynecologists, otolaryngologists, surgeons, urologists, radiotherapists and medical oncologists) regarding the approach to sexual health during consultations with oncology patients.

## Materials and methods

This is a descriptive, cross-sectional, observational study that included postgraduate medical professionals and oncologic and non-oncologic specialists who deal with oncologic patients, analysing the approach to sexual health and its relevance in daily practice in relation to different parameters.

### Target population

Medical professionals related to oncology patients (gynecologists, otorhinolaryngologists, surgeons, urologists, radiotherapists and medical oncologists). Prior to completing the survey each participant accepted an informed consent. No data that could identify the participants were used and all information was handled anonymously.

### Survey design and development

The survey was designed ad hoc for this study, with the purpose of comprehensively exploring the practices, perceptions and barriers in the approach to sexual health by medical professionals caring for oncology patients, capturing a wide range of relevant aspects in this context.

To ensure the relevance and comprehensibility of the questions, the design of the questionnaire was based on a comprehensive review of the existing literature on sexual health in oncology, which allowed us to identify the main issues and barriers in this area. The items were selected for their ability to capture critical information and were validated by a panel of oncology and sexual health experts.

A pilot test was conducted with a small group of medical professionals (*N* = 10), who provided feedback on the clarity and usability of the questionnaire. Based on their feedback, adjustments were made to simplify the wording, improve the response scale and add items addressing specific barriers.

The questions were grouped into different domains:

Demographic and professional information: Questions on age, gender, medical specialty and years of experience to characterize the study population.

Addressing sexual health: Questions designed to understand how and how often professionals address sexual health in their consultations, including whether it is discussed at the first consultation and the frequency of these discussions according to the type of treatment (curative, palliative and so on).

We also inquired about barriers to addressing sexual health: Sections dedicated to identifying the main limitations perceived by professionals to discuss sexual health, such as lack of time, training or discomfort with the topic; and about the need for training of the surveyed physicians: Questions on current knowledge and perceived need for additional training in sexual health.

Each question offered several response options, allowing participants to select the one that best represented their experience or perception. Both multiple-choice and Likert-scale questions were included to assess the frequency and degree of agreement with specific statements.

The informed consent phase precedes the survey itself, ensuring that all participants are duly informed about the purpose of the study, the confidentiality of their responses and their right to withdraw at any time without consequences.

The questionnaire was created using the SurveyMonkey server, an online platform that allows the distribution and collection of responses anonymously and securely. The survey was disseminated among groups of specialists and postgraduate professionals working with oncology patients, using various media such as e-mails and professional groups in social networks, to reach a representative sample of the target population.

A pilot test was conducted with a small group of medical professionals to ensure the clarity of the questions and the usability of the instrument. The comments and suggestions collected during the pilot test were used to make final adjustments to the questionnaire before its definitive implementation.

Data analysis: Univariate and multivariate analysis was carried out to examine the data of the variables in the population according to age, gender, specialty, experience and degree of training. Univariate comparisons were performed using different appropriate statistical tests. A significance level of *p* < 0.05 was established for all analyses performed. Data analysis was performed using Statistical Package for the Social Sciences 25.

### Ethical aspects

This study was approved by the Ethics Committee Hospital de Clínicas and was conducted following international ethical regulations for biomedical research, in accordance with the ‘MERCOSUR Norms on regulation of clinical studies’, the ‘Declaration of Helsinki’ and the research regulations endorsed by the National Ethics Commission in 2019. All participants had to accept informed consent prior to completing the questionnaires.

## Results

Of a total of 133 respondents, 61.7% (*N* = 82) were women. In relation to age group, 45.1% were in the 25–35 years age group. Regarding educational level, 66.2% had a specialist degree, in contrast to 33.8% who were postgraduate students or residents (Table 1).

In terms of specialty, 33.9% identified themselves as medical oncologist or radiation oncologist, while 66.1% belonged to other specialties, as detailed in Table 2.

### Addressing sexual health in clinical practice

Of the total respondents, 30.1% responded that they never or rarely discuss sexual health with their patients, 31.6% do so less than half of the time and 13.5% do so frequently. Likewise, more than half of the respondents (51.1%) rarely or never ask about this topic at the first contact with the patient and only 8.3% always or almost always do so.

When asked how often the topic is spontaneously addressed, 37.6% indicated that it is addressed less than half of the time, 35.3% responded rarely or never and only 6.8% answered frequently or always.

If we look at how often the patient’s partner is included in the discussion of sexual health, it was found that 44.4% never or rarely include it, 30.1% do so in less than half of the cases and only 6.8% in more than half of the cases.

According to the type of treatment patients receive (palliative symptomatic, curative or with the aim of prolonging survival), the majority of respondents answered that they discuss sexual health in those under treatment with curative intent (77.4%), followed by treatments that seek to prolong survival (17.3%), while only 5% do so in patients with palliative symptomatic treatment.

Regarding the influence of the patient’s gender, 49.6% indicated that gender would have no impact on the approach. When asked specifically about which gender is most frequently addressed, the responses showed a balance for both genders.

Regarding the age range in which the most counseling is performed, 37.6% do it between 36 and 50 years old, 29.3% between 51 and 65 years old, 25.6% between 16 and 35 years old and only 7.5% performed it in patients older than 66 years old.

### Sexual health approach according to type of cancer

The frequency with which sexual health is addressed varies according to the type of cancer. For breast cancer, 11.3% address it in more than half of the cases. For prostate cancer, 12.8% address sexual health in more than half of the cases. For both rectal and colon cancer, 1.5% and 3.8%, respectively, address it in half of the cases, while 35.3% never do so. In patients with cervical cancer, 7.5% do so in half of the cases, with 18.5% never addressing sexual health in these patients. In tumours of ENT and bladder origin, sexual health is the least frequently addressed (Table 3).

Looking at the data obtained globally, sexual health is most frequently discussed in patients with prostate, cervical and breast cancer. In contrast, it is observed that the discussion of sexual health is less frequent or rarely addressed in patients with tumours in areas such as ENT, bladder and colorectal.

### Barriers to addressing sexual health

Among the main limitations in the approach to sexual health, 46.6% attributed it to lack of training, followed by lack of time during the consultation or their own or the patient’s discomfort (Table 4).

### Knowledge of oncologic sexuality and need for training

10.5% indicated that they have sex training frequently or always, 12% in more than half of the cases, while 36.4% in less than half of the cases and 24.8% refer that they rarely or never have tools.

Regarding the need for training for sexual health counseling, 92.5% indicated that there is a need for tools. When mentioning the need for tools for sexual health counseling, reference is made to a broad spectrum of resources that can facilitate addressing these issues in an effective and empathetic manner, including:

- Technological tools: Digital learning platforms, mobile applications for sexual health education and databases with updated information on sexuality and cancer.
- Training materials: Clinical guidelines, best practice manuals, online training modules and face-to-face workshops that specifically address sexuality in the context of oncology.
- Specialised counseling: Access to consultations with experts in sexual health and oncology to discuss complex cases or specific questions, and the possibility of referring patients to these specialists when more detailed care is required.
- Patient support resources: Informational brochures, web pages with educational content and helplines that professionals can offer patients to complement the face-to-face consultation.

According to the age range of the respondents regarding the need for sexual health training, for the 25–35 age group, 95% considered that it is necessary. For the 35–55 years age range, 93% stated that training is required and over 55 years of age, 80% considered that sexual health training is necessary, this difference was not statistically significant (*p* = 0.139).

According to the gender of the respondents, 95.1% of the female gender considered the need for sexual health training, while 88.2% of the male gender considered it necessary, this difference not being statistically significant (*p* 0.143).

In relation to the specialties surveyed, more than 90% of surgeons, gynecologists, otorhinolaryngologists and other medical specialties consider that training in sexual health is necessary. However, among urologists, this percentage is reduced to 66%. Ninety-seven percent of medical oncologists expressed the need for training, and this difference proved to be statistically significant (*p* 0.035), (Table 5).

According to the degree of training attained, 97.8% of the professionals who have not yet completed their specialty recognize the importance of receiving training in sexual health, while among those who have already completed their studies, 89.8% share this perception. Although there is a tendency for postgraduates to see a greater need for training, this disparity does not reach statistical significance (*p* = 0.098).

Those professionals with less than 5 years of experience and up to 10 years, 95.8% and 91.3%, respectively, agree on the need for tools to address sexual health, while 87% of those with more than 10 years of experience consider it necessary, the difference not being statistically significant (*p* = 0.255).

## Discussion

This study set out to investigate how sexual health is addressed in the oncologic context by examining the practices of both medical oncologists and other specialists involved in the care of cancer patients. Interestingly, it was observed that the majority of respondents were not medical oncologists; out of a total of 133 professionals surveyed, only 44 were medical oncologists, while 89 belonged to other specialties. This diversity in the profile of respondents underscores the importance of a multidisciplinary approach to sexual health management within oncology care, highlighting the need to integrate diverse perspectives and competencies to effectively address this critical aspect of patient well-being.

Aspects such as type of cancer, patient gender, treatment goals, existing barriers and the need for specialised training were evaluated. Given the growing evidence of the significant impact that sexual health has on patients’ quality of life, this analysis also seeks to highlight the importance of effective communication between patients and health professionals.

This study reveals that most health professionals rarely or never address sexual health in oncology consultations, coinciding with previous findings highlighting insufficient communication about sexual health among patients and professionals [14, 11]. Previous research, such as Stead *et al* [15] involving physicians and nurses, Park *et al* [16] among internists and studies focused on gynecologic oncologists [17], confirm this trend. In addition, reviews in the United Kingdom [18] and work by Haboubi and Lincoln [19] highlight the acknowledged existence of the problem, but the limited discussion is often attributed to a lack of time and adequate training in this area.

However, multiple studies show the dissatisfaction and need of patients for information on sexual health consequences at the time of cancer diagnosis and treatment initiation, where health professionals prioritize other symptoms and complications [11, 19, 20].

The present study shows a predominant tendency to address sexual health in patients undergoing treatment with curative intent (77.4%), comparatively less in those receiving life-prolonging treatments (17.3%) and minimally in patients undergoing palliative treatment (5%). This distribution is consistent with international research findings, such as the study by Krouwel *et al* [12], which shows significant variability in the approach to sexual health based on the purpose of treatment. However, it is relevant to highlight that there is a wide range of studies focused on the importance of sexual health during the advanced stages of cancer, underlining its essential contribution to quality of life. In the context of palliative care, intimacy and physical contact take on critical importance [11, 20–23], reinforcing the need for a comprehensive approach that incorporates sexual health as a key component of patient well-being.

In addition to sexual function, it is crucial to consider the affectivity and the need for intimacy of oncology patients. Cancer and its treatments can have a profound impact on couple relationships, generating anxiety and intimacy difficulties. Healthcare professionals should be trained to address these issues, supporting both patients and their partners in the process of adapting to changes in their sexual and emotional lives. Involving the partner in discussions about sexual health can strengthen emotional support and improve the quality of life for both partners. Couple-centered care allows for a more complete understanding of challenges and needs, facilitating a more holistic approach to cancer care [11, 20, 22].

A pilot study of palliative care providers’ attitudes and beliefs toward discussing sexual health in cancer patients highlights the infrequent attention to this critical aspect of health. In the anonymous survey conducted, 69% of palliative care professionals indicated that they rarely or never discussed sexual health with their patients, primarily attributing this responsibility to oncologists. The main reasons for avoiding the topic included the patient not initiating the conversation, lack of time and the presence of third parties. Despite acknowledging the need for more training and finding the printed material helpful, most admitted to not routinely addressing sexual health disturbances. This finding underscores the urgency of providing additional training and establishing routine screening for sexual health, which could facilitate the integration of sexual health into the overall care of the cancer patient, including in the palliative care setting [24].

In this context, the lack of sexual health dialogue at these stages can increase anxiety and decrease quality of life, underscoring the need for more comprehensive care [25–27].

Regarding barriers to addressing sexual health; lack of training (46.6%), limited time in consultations (39.8%) and discomfort between patients and professionals (34.6%) emerged as the main barriers to discussing sexual health, which is consistent with what has been previously reported internationally [16]. Barriers vary from patient characteristics, such as age or partner status, to the dynamic with the professional, where fear of mutual embarrassment may inhibit conversation. In addition, information overload following diagnosis can make it difficult for patients to address sexual concerns [16–18]. These barriers underscore the need for strategies to improve sexual health communication in oncology.

Sexual health counseling in our study decreased with the age of the patients, concentrating mainly between 36 and 50 years of age (37.6%), and decreasing markedly in those over 66 years of age (7.5%). This pattern reflects similar trends in the literature, where Krouwel *et al* [12] highlight a greater discussion in younger patients. The authors identify advanced patient age as a significant barrier to discussing sexual function and suggest that this phenomenon may be due to the assumption by oncologists that older patients do not maintain sexual activity. Such an assumption underscores a critical area for improvement in clinical practice, highlighting the importance of challenging stereotypes related to sexual health in old age and ensuring that patients’ sexual health needs are addressed in a comprehensive manner and without age-related bias. In this context, a recent systematic review has shown that sexual health is a crucial component of subjective well-being in older people, ranging from age 40 to 90 years or older [25].

In addition, age impacts both genders; older women experience more risk of sexual dysfunction, particularly those surviving tumours of gynecologic origin, compared to younger patients [26, 27]. For the male gender, age is also an important factor, being a determinant in the recovery of sexual function after radical prostatectomy, with patients younger than 50 years showing better outcomes compared to those older than 70 years, who face higher risks and worse functional outcomes [28].

The relevance of these findings underscores the importance of not allowing patient age to limit the approach to sexual health in oncology, recognising its importance for quality of life at any age.

The present analysis indicates variability in the approach to sexual health according to the type of cancer, with greater frequency in genitourinary cancers such as prostate (12.8%, in more than half of the cases), breast (11.3%, in more than half of the cases) and cervix (7.5%, in more than half of the cases), and less in cancers of areas such as colorectal, ENT and bladder. This pattern suggests a perception of lower relevance of sexual health in non-genital cancers, which is consistent with other studies emphasizing the prevalence and impact of sexual dysfunction on the quality of life of survivors, particularly in these types of cancer [29]. These findings underscore the need for comprehensive and personalised sexual health management for all cancer patients, emphasizing the importance of overcoming communication barriers and providing adequate support across a broader spectrum of malignancies.

In this sense, although colorectal cancer is the second most frequent tumour in Uruguay for both sexes [30], the present study revealed that 35% of the professionals never or rarely discuss sexual health in these cases, despite the known post-treatment sexual dysfunctions such as erectile dysfunctions and alterations in body image due to colostomies [10, 31–33]. Similarly, in patients with bladder cancer, radical cystectomy affects sexual health [34, 35]; however, 36.1% of respondents rarely or never addressed this issue in the consultation. In relation to otorhinolaryngological (ENT) tumours, 48.9% of professionals rarely or never address sexual health, despite the fact that treatments can significantly alter body image and self-esteem, profoundly affecting intimacy [36–38]. In this regard, a study revealed that 5 years after head and neck cancer diagnosis, most survivors experience significant decreases in sexual quality of life, affecting 78.8% of men and 79.2% of women. Dissatisfaction with sexual frequency and decreased sexual desire were associated with radiotherapy and chemotherapy treatments, respectively. These findings highlight the urgency of addressing the sexual health of survivors, in line with our observation of the infrequent discussion of sexual health in patients with ENT tumours [39]. In addition, many of these tumours are associated with the sexually transmitted Human Papillomavirus, which can intensify feelings of guilt and responsibility, further impacting patients’ intimacy [40, 41].

The study shows that 44.4% of professionals never or rarely include partners in discussions about sexual health, and only 6.8% involve the partner in more than half of the cases despite the fact that it is known that sexual alterations impact not only the patient but also his or her partner, an aspect confirmed by studies indicating the profound effect of cancer on intimacy and sexual health of couples [42–46].

The majority of respondents in the present study acknowledged a lack of sexual health training, with less than 20% reporting having the necessary tools frequently or in more than half of the cases. Comparative studies in Saudi Arabia [47] and France [48] also reveal deficiencies in knowledge about oncosexuality, highlighting barriers such as religious influence and demand for specific training. Abdolrasulnia *et al* [49] identified similar gaps among primary care physicians and gynecologists, pointing to time constraints and lack of effective therapies as additional barriers. The need for educational programs and ongoing training in oncosexuality is evident to improve sexual health care, suggesting integration of this training with oncologic treatment to overcome the time barrier.

The vast majority of professionals in the present study (92.5%) recognize the need for training in sexual health, reflecting a significant lack of knowledge and tools to address this issue, similar to that reported by Krouwel *et al* [12] and in line with the demand for more information from patients, as shown in studies conducted by Almont *et al* [48] and Wazqar *et al* [47]. The gap between the perceived need for training among urologists (66%) and oncologists (97%) suggests variations in perceived competence according to specialty, despite the high demand for sexual counseling in patients, particularly those with prostate cancer [50]. The data also indicate that there are no significant differences in the perception of the need for training based on gender, age or years of experience of the professionals (*p* > 0.05). Previous studies, such as those by Julien *et al* [51] and Leonardi *et al* [52], highlight how prior experience and training can influence practitioner comfort in addressing sexual health, underscoring the importance of integrating sexual health education into the medical training curriculum to improve oncology care.

Our work is pioneering in Uruguay, being, to the best of our knowledge, the first study that comprehensively addresses the treatment of sexual health in the oncologic context from the perspective of health professionals. It meets the proposed objectives, but recognizes certain limitations.

A significant limitation of this study is its cross-sectional design, which, while providing a valuable snapshot of current practices and perceptions of sexual health in oncology at the national level, does not facilitate analysis of changes over time or determination of causalities. Despite the fact that the specifically designed questionnaire captured a broad spectrum of relevant aspects of sexual health management, the response rate and representation of various medical specialties could be improved to provide a more complete and varied perspective.

Another limitation is the absence of questions specifically aimed at capturing the experiences of LGTBIQA+ patients in our analysis, which limits the understanding of sexual and gender diversity in this context. Although this exclusion responds to initial methodological decisions, it highlights the importance of expanding future research to explicitly include sexual and gender diversity, thus ensuring a more inclusive and representative approach to sexual health in oncology.

Among its strengths, it highlights the multidisciplinary approach, which encompasses a wide range of specialties involved in oncologic care, reflecting the complexity and the need for comprehensive treatment for sexual health in these patients. The methodology applied, which includes the creation of a specific questionnaire and detailed analysis of the responses, provides valuable insights into current practices, barriers and training needs, emphasizing the urgency of developing effective educational and communication strategies to improve the quality of oncology care.

## Conclusion

Despite the increased survival of cancer patients and the relevance of sexual health in their quality of life, there is a notable gap in the approach to sexual health by both oncologists and non-oncologists. The main barrier identified is the lack of specific training in sexual health, complemented by time constraints and discomfort in discussing the topic and particularities of the patient and their disease. This study revealed that discussion of sexual health is more frequent in patients with curative treatments, while it is minimized in the palliative context. The inclusion of the partner in these discussions emerges as a crucial component, which can strengthen emotional support and improve the quality of life related to sexual health.

A significant finding is the almost unanimous recognition of the need for training in sexual health, suggesting a widespread willingness to improve this area of oncologic care. The demand for training transcends gender, age and specialty, although a particularly pressing need is noted among oncologists. This study underscores the importance of integrating sexual health education into the medical training curriculum and clinical practice to facilitate effective and empathetic communication with patients and their partners about the sexual consequences of cancer and its treatments. The implementation of educational strategies and the development of practical tools for professionals are essential steps towards a more holistic cancer care that not only focuses on patients’ survival, but also on their quality of life and that of their partners, proactively and sensitively addressing sexual health as an integral component of overall health.

## Conflicts of interest

The authors have no conflicts of interest.

## Funding

The study was not financed.

## Figures and Tables

**Table 1.** Characteristics of the population.

Epidemiological variables	*N* (%)
Age range 25–35 35–55 More than 55	60 (45.1) 58 (43.6) 15 (11.3)
Gender Female Male	82 (61.7) 51 (38.3)
Specialty Medical oncologist/radiotherapist Non-oncologist	45 (33.9) 88 (66.1)
Degree of training Received Resident/Postgraduate	88 (66.2) 45 (33.8)
Years of experience Less than 5 Between 5 and 10 More than 10	71 (53.4) 23 (17.3) 39 (29.3)

**Table 2.** Distribution of specialties.

Specialists	*N* (%)
Medical Oncologist	44 (33.1)
Gynecologists	24 (18)
Urologists	15 (11.3)
ENT	14 (10.5)
General Surgeons	13 (9.8)
General Practitioner	9 (6.8)
Palliative Care Physician	7 (5.3)
Internist Physician	5 (3.8)
Family Physician	1 (0.8)
Radiation Oncologist	1 (0.8)

**Table 3.** Sexual health approach according to types of cancer.

	Mama *N* (%)	Prostate *N* (%)	Colon *N* (%)	Straight *N* (%)	Cervix *N* (%)	ENT *N* (%)	Bladder *N* (%)
Frequently/always	**22 (16.5)**	**30 (22.6)**	8 (6)	7 (5.3)	**28 (21.1)**	8 (6)	12 (9)
In more than half of the cases	15 (11.3)	17 (12.8)	6 (4.5)	5 (3.8)	14 (10.5)	4 (3.0)	4 (3.0)
In half of the cases	17 (12.8)	11 (8.3)	5 (3.8)	2 (1.5)	10 (7.5)	-	6 (4.5)
In less than half of the cases	25 (18.8)	16 (12)	30 (22.6)	33 (24.8)	26 (19.5)	20 (15)	27 (20.3)
Never/rarely	29 (21.8)	24 (18)	**47 (35.3)**	**47 (35.3)**	25 (18.8)	**65 (48.9)**	**48 (36.1)**
Not applicable	25 (18.8)	35 (26.3)	37 (27.8)	39 (29.3)	30 (22.6)	36 (27.1)	36 (27.1)

**Table 4.** Barriers to addressing sexual health.

	*N* (%)
Lack of training	62 (46.6)
Lack of time	53 (39.8)
Patient and/or disease characteristics	44 (33.1)
Self/patient discomfort	46 (34.6)

**Table 5.** Need for training in sexual health according to specialties.

Specialty	Yes (%)	No (%)
Medical Oncologist	43 (97.7)	1 (2.3)
ENT	13 (92.9)	1 (7.1)
Surgeon	12 (92.3)	1 (7.7)
Gynecologist	22 (91.7)	2 (8.3)
Urologist	10 (66)	5 (33.3)
Family Physician	1 (100)	0
General Practitioner	9 (100)	0
Internist Physician	5 (100)	0
Palliative Care Physician	7(100)	0
Radiation Oncologist	1 (100)	0
